# Low-Temperature Growing Anatase TiO_2_/SnO_2_ Multi-dimensional Heterojunctions at MXene Conductive Network for High-Efficient Perovskite Solar Cells

**DOI:** 10.1007/s40820-020-0379-5

**Published:** 2020-01-31

**Authors:** Linsheng Huang, Xiaowen Zhou, Rui Xue, Pengfei Xu, Siliang Wang, Chao Xu, Wei Zeng, Yi Xiong, Hongqian Sang, Dong Liang

**Affiliations:** 1 National Engineering Research Center for Agro-Ecological Big Data Analysis and Application, School of Electronics and Information Engineering, Anhui University, No. 111 Jiulong Road, Hefei, 230601 People’s Republic of China; 2 Science and Technology Institute, Hubei Key Laboratory of Biomass Fibers and Eco-Dyeing and Finishing, Wuhan Textile University, Wuhan, 430073 People’s Republic of China; 3 School of Physics and Technology, MOE Key Laboratory of Artificial Micro- and Nano-Structures and Center for Electron Microscopy, Wuhan University, Wuhan, 430072 People’s Republic of China; 4 Department of Physics, King’s College London, The Strand, London, WC2R 2LS UK

**Keywords:** In situ fabrication, Multi-dimensional heterojunction, Oxygen vacancy scramble effect, Electron transport layer, Perovskite solar cells

## Abstract

A multi-dimensional conductive heterojunction structure, composited by TiO_2_, SnO_2_, and Ti_3_C_2_T_*X*_ MXene, is facilely designed and applied as electron transport layer in efficient and stable planar perovskite solar cells. Based on an oxygen vacancy scramble effect, the zero-dimensional anatase TiO_2_ quantum dots, surrounding on two-dimensional conductive Ti_3_C_2_T_*X*_ sheets, are in situ rooted on three-dimensional SnO_2_ nanoparticles, constructing nanoscale TiO_2_/SnO_2_ heterojunctions. The fabrication is implemented in a controlled low-temperature anneal method in air and then in N_2_ atmospheres. With the optimal MXene content, the optical property, the crystallinity of perovskite layer, and internal interfaces are all facilitated, contributing more amount of carrier with effective and rapid transferring in device. The champion power conversion efficiency of resultant perovskite solar cells achieves 19.14%, yet that of counterpart is just 16.83%. In addition, it can also maintain almost 85% of its initial performance for more than 45 days in 30–40% humidity air; comparatively, the counterpart declines to just below 75% of its initial performance.

**Electronic supplementary material:**

The online version of this article (10.1007/s40820-020-0379-5) contains supplementary material, which is available to authorized users.

## Highlights

- Nanoscale multi-dimensional heterojunctions in situ grow at the edge of two-dimensional MXene conductive network.
- A controlled anneal procedure in 150 °C is for preparing anatase TiO_2_/SnO_2_ heterojunctions with oxygen vacancy scramble effect.
- The perovskite solar cells achieve high power conversion efficiency and high moisture-resistance stability.

**Electronic supplementary material:**

The online version of this article (10.1007/s40820-020-0379-5) contains supplementary material, which is available to authorized users.

## Graphical abstract

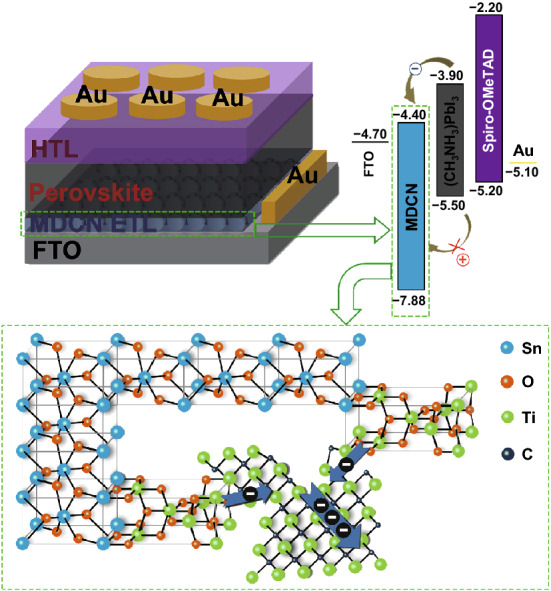

## Introduction

The organic–inorganic hybrid lead halide perovskite solar cells (PSCs) have shown tremendous promotion of power conversion efficiency (PCE) from 3.8% to a certified 25.2% in less than a decade, with fascinating industrial potentials of low cost and simple solution process [1, 2]. As an important functional interlayer in PSC device, the electron transport layer (ETL) plays an important role. In a conventional upright structure in PSCs, the light absorption property of perovskite layer can be affected by the optical transmittance property of ETL by reducing optical energy loss [3]. The charge carriers in device can be regulated by the semiconductive energy-level property of ETL, through selectively transporting electrons and blocking holes from the adjacent perovskite layer [4]. Further, the amount of photon-excited carrier in perovskite layer can be restricted by the ETL, because the crystal size of perovskite can be affected by the hydrophobicity of the below ETL in preparation [5, 6]. Therefore, in order to achieve high PCE, the ETL is required to be facially designed and fabricated based on its material and nanostructure to acquire high optical transmittance, matched semiconductive band, suitable material, and morphological features [7–9].

The SnO_2_ has been demonstrated one of the superior electron transport materials (ETMs) in PSCs by harvesting the highest PCE of 21.6% to this day, because of its better optical and electric properties, energy band with respect to perovskite layer, and simple solution preparation in low-temperature anneal method, which can be potentially applied in flexible devices [10–12]. However, it still suffers from some drawbacks, such as poor wetting property, weak transmittance, and low conductivity [12]. The doping in solution preparation is one of simple effective methods to improve ETM, exhibiting a fascinating potential in large-scale industrial application. For the SnO_2_-based ETL, the anneal temperature is generally below 185 °C. On the basis of the solution preparation in low-temperature anneal, the energy band alignment can be engineered by ion doping of Y^3+^, Sb^3+^, Li^+^, Ti^4+^, Nb^5+^, and ethylene diamine tetraacetic acid in Sn ion or SnO_2_ solution [13–17], and the electric conductivity can be facilitated by Li^+^-, Mg^2+^-, and Sb^3+^-based p-type dopant [15, 18]. The ETL/perovskite interface can be improved by passivating the bulk defects and mitigating the interfacial charge recombination with Cl^−^ and Y^3+^ dopant [3, 13]. Hereof, the Ti-based dopant is attractive, because the TiO_2_, as a most widely used ETM, is demonstrated to form effective bilayered TiO_2_/SnO_2_ heterojunction, achieving the highest PCE of 21.4% recently [19, 20]. However, the anneal temperature as high as 450–500 °C is generally necessary in these reports for forming effective TiO_2_ phase in anatase lattice [21]. On the basis of a low-temperature anneal at 75 °C, the TiCl_4_ solution can be translated into needle-like TiO_2_ layer in rutile phase when it is heated by covering a soft film, yet the PSC based on this TiO_2_ ETL just shows a PCE of 17.09% [22]. In a low-temperature anneal at 120 °C, a TiO_2_ layer can be also formed based on aqueous TiCl_4_. Although the resultant TiO_2_/SnO_2_ composite can contribute a maximum PCE of 21.27% [23], this PCE is still restricted by the amorphous phase of TiO_2_ here [17]. Therefore, it is still deserved to adjust the Ti component on its lattice phase and nanostructure for gaining higher PCE of device.

The 2D materials and their tailored products, such as black phosphorene and 0D graphene quantum dot (QD), have been employed as dopants to modify the SnO_2_ ETLs in PSCs on the electrical conductivity, energy-level properties, and reducing the surface defects and surface electron traps [24, 25]. As a new group of 2D materials, the MXene is constructed of M_*N*+1_X_*N*_T_*X*_ structure, where the M represents an early transition metal such as Ti, and the X representing C and/or N element, and the T_*X*_ meaning the terminal functional groups [26]. With its excellent metallic electrical conductivity, hydrophilic surface, and pseudocapacitance properties, the MXene has been explored as electrode materials for solar cells [27–29], zinc-ion capacitors [26, 30], supercapacitors [31], or sensors [32]. In PSC application, it has also been reported that the PCE can be enhanced on the basis of the improved functional layers with MXene dopant. With Ti_3_C_2_T_*X*_ MXene-doped perovskite layer, the crystal size of CH_3_NH_3_PbI_3_ and charge transfer can be both increased [28]. Also, with Ti_3_C_2_ MXene-doped SnO_2_ ETL, the charge-transfer paths, electron mobility/extraction can be all improved in device [29]. Further, it is potential to advance the MXene-doped SnO_2_ ETL by introducing the TiO_2_ in suitable crystal phase for forming effective heterojunction structure.

By oxidation in a hydrothermal route and subsequent hydrazine hydrate reduction, a Ti^3+^-doped TiO_2_ in rutile lattice can be composited with 2D Ti_3_C_2_ MXene sheets, using MXene itself as Ti source [33]. By partial oxidation hydrothermal route, the 2D anatase TiO_2_ can be in situ grown on Ti_3_C_2_ for the interface with minimized defects [34]. Through in situ hydrolysis and heat treatment, the 3D anatase TiO_2_ nanoparticles can be decorated on Ti_3_C_2_ MXene [35]. Based on these hydrothermal methods, the nanoparticle size of anatase TiO_2_ is about 30 nm, and the TiO_2_ is assembled on the surface of Ti_3_C_2_T_*X*_ MXene nanosheets [36–39]. Then, Schottky junction can be formed at the interface of TiO_2_/MXene for promoting the carrier separation by hole trapping effect [33, 34]. Besides, with a method of high-energy ball mill, the anatase TiO_2_ can be also generated on carbon nanosheets, and the raw 2D Ti_2_CT_*x*_ MXene serves as Ti and carbon source [40]. On the other hand, the Ti-based oxide can be also grown at MXene in air environment, because of its unstable property. By annealing in 250 °C in air, the anatase TiO_2_ can be formed at MXene [41]. Among these methods, the anneal is relatively economical. The resultant MXene-based anatase TiO_2_ is potential to promote the PCE of SnO_2_-based PSCs by forming TiO_2_/SnO_2_ heterojunction and conductive MXene pathways, because it is reported that the stack of MXene sheets can be prevented by the SnO_2_ QDs as “spacer”, and the highly conductive Ti_3_C_2_T_*X*_ MXene can provide efficient pathways for fast transport of electrons [42]. However, the annealing temperature for MXene-based anatase TiO_2_ is higher than that for SnO_2_ in low-temperature fabrication. Therefore, it is desired to improve this low-temperature anneal method for economical application.

Herein, the TiO_2_ QDs in anatase phase are composited with SnO_2_ ETM by adopting MXene in SnO_2_ solution with a controlled anneal method in 150 °C low temperature, implemented in air and N_2_ atmospheres. The nanoscale TiO_2_/SnO_2_ heterojunctions are formed, and the connectivity of ETL is enhanced as the 2D MXene acting conductive bonded bridges, contributing a multi-dimensional conductive network (MDCN) structure for ETM. In addition, the crystal size of upper perovskite layer is enlarged and the ETL/perovskite interface is improved. With the optimal MDCN ETL, a champion PCE of 19.14% of PSC is achieved. The PSC also exhibits negligible hysteresis and higher moisture-resistance stability even after 45 days at room temperature, compared to the controlled devices.

## Experimental Section

### Materials and Reagents

Formamidinium iodide (FAI, > 99.5%), methylammonium chloride (MACl, > 99.5%), methylammonium bromine (MABr, > 99.5%), and 2,2′,7,7′-tetrakis-(N,N-dip-methoxyphenylamine) 9,9′-spirobifluorene (Spiro-OMeTAD, > 99.5%) are purchased from Lumtec, Taiwan, China. The SnO_2_ colloid solution (15 wt%), isopropanol (IPA, > 99.5%), and acetonitrile (> 99.8%) are obtained from Alfa Aesar. N,N-dimethylformamide (DMF, > 99.8%) and dimethyl sulfoxide (DMSO, > 99.9%) are achieved by Sigma-Aldrich. Li-bis(trifluoromethanesulfonyl)imide (Li-TFSI), 4-tert-butylpyridine (tBP), lead(II) iodide (PbI_2_, 99.9%), lithium fluoride (LiF, 99.9%), ethanol, and acetone are received from Aladdin. The Ti_3_AlC_2_ powders are obtained from Jilin 11 Technology Co., Ltd. All the chemicals and solvents reagents are directly used without further purification.

### Preparation of MXene and PSCs

#### MXene Preparation

The MXene nanosheet solution was prepared by the following manufacturing procedures [31]. After the deionized water was added to a hydrochloric acid solution to prepare 10 mL 9 M hydrochloric acid, 0.5 g LiF powder was slowly added to this hydrochloric acid solution and then was stirred for 10 min. Then, 0.5 g Ti_3_AlC_2_ was slowly added into the above-mixed solution and then stirred at 35  °C for 24 h. Next, the residue was washed through centrifugation with deionized water at 3500 rpm for several times until pH > 6. Then, the product was sonicated under ice bath in an Ar atmosphere for 30 min. Finally, the product was centrifuged for 30 min at 3500 rpm to collect the MXene nanosheets. The concentration of resultant MXene was adjusted as 9 mg mL^−1^.

#### MDCN Precursor Preparation

A 2.67 wt% SnO_2_ solution was obtained by diluting SnO_2_ colloid solution with deionized water. Then, the SnO_2_-MXene mix precursor was prepared by adding 0.02 wt% MXene into this SnO_2_ solution. The mix precursor was ultrasonically treated over 30 min at room temperature before use so as to acquire more tiny-MXene flakes.

#### Device Fabrication

**Fig. 1:**
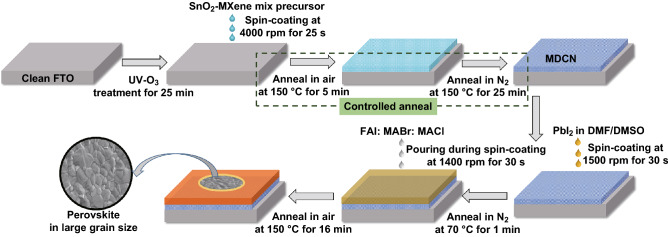
Fabrication process for perovskite layer with MDCN ETL

The fabrication process for perovskite layer with MDCN ETL is sketched in Fig. 1. The fluorine-doped SnO_2_ (FTO) glasses as substrates were ultrasonically cleaned with detergent, deionized water, acetone, isopropanol and ethanol for 20 min in sequence. After the substrates were treated with UV-O_3_ for 25 min, the SnO_2_-MXene mix precursor was spin-coated on the substrates at 4000 rpm for 25 s and then was controlled annealed at a low temperature of 150 °C. This controlled annealing process is operated in air for 5 min and then in N_2_ atmosphere for 25 min in a glove box, resulting the MDCN ETL. For comparison, the annealing process is just operated in air for entire 30 min, resulting in the pristine SnO_2_ ETL.

The perovskite layer with (FAPbI_3_)_0.97_(MAPbBr_3_)_0.03_ was fabricated on the ETL by a two-step deposition strategy as described in our previous work [21, 43]. In belief, 1.3 M PbI_2_ in the mixture solvent of DMF/DMSO (9.5:0.5, by volume) was spin-coated onto the ETL at 1500 rpm for 30 s and then annealed at 70 °C for 1 min in N_2_ atmosphere in a glove box. After the PbI_2_ layer has been cooled down, 50 μL mixture solution of FAI/MABr/MACl (1:0.15:0.25, molar ratio, in 1 mL IPA) was poured on the PbI_2_ layer during the spin-coating process, and then the substrate was continuously spun at 1400 rpm for 30 s. Then the resultant substrates were transferred into ambient air in 40–60% humidity and were heated on a hot plate at 150 °C for 16 min. After the perovskite layer was cooled to the room temperature, the perovskite crystals with large grain size was generated, which will be discussed in the following sections. Then, the precursor for hole transporting layer (HTL) was coated on the perovskite film at 4000 rpm for 20 s, resulting a HTL. Here, the HTL precursor contained 85.8 mg spiro-OMeTAD, 19.5 μL Li-TFSI (520 mg in 1 mL acetonitrile), 35.5 μL tBP and 8 μL FK209 (400 mg in 1 mL acetonitrile) in 1 mL chlorobenzene. Finally, an Au counter was fabricated on the HTL as top electrode by thermal evaporation in high vacuum.

### Characterization

The morphology is analyzed by a scanning electron microscopy (SEM, Hitachi S-4800), transmission electron microscopy (TEM, JEOL JEM-2010), high-resolution TEM (HRTEM, JEOL-2010), and atomic force microscopy (AFM, SPM-9700). The energy-dispersive spectroscopy (EDS) was conducted on a SEM (S-4800). The photovoltaic properties are characterized by current density–voltage (*J* − *V*) measurements under one sun simulated illumination (AM 1.5G, 100 mW cm^−2^) in air. The active area of samples is fixed as 0.1 cm^2^, which is confined by the fixed area of Au electrode, and the scan rate is set as 0.15 V s^−1^ in *J* − *V* measures. The electrochemical impedance spectroscopies (EIS) are measured with a whole PSC device and are carried out with frequencies ranging from 100 kHz to 0.01 Hz at 0.74 V in dark. The ultraviolet–visible absorbance and transmission spectra were measured by an ultraviolet–visible spectrophotometer (UV–Vis, U3900H). The crystal, chemical state, and energy-level properties are analyzed with X-ray diffraction (XRD, SmartLab, 9 kW), X-ray photoelectron spectroscopy (XPS, ESCALAB 250Xi), and ultraviolet photoelectron spectroscopy (UPS, ESCALAB 250Xi), respectively. The external quantum efficiency (EQE) was measured by an Enli Tech EQE measurement system (Taiwan, China) with a whole PSC device, and light intensity was calibrated by using a standard single crystal Si photovoltaic cell. The samples for SEM, XPS, UV–Vis absorption, and transmission spectra are fabricated on FTO substrates with the dip-coating method described in experimental part. The steady-state photoluminescence (PL, QM400-TM) is measured at excited laser at 470 nm, and the time-resolved photoluminescence (TRPL) is recorded by the pulsed nitrogen/dye laser (QM400, Photo Technology International, USA). The transient photovoltage (TPV) and transient photocurrent (TPC) measurements are carried out with the same test device structure and the same equipment as those for *J* − *V* measures, and the incident time of transient light is fixed as 0.1 s, which is generated by a shutter on the sun simulator. The space-charge-limited currents (SCLCs) are measured with a test device structure of Au/ETL/FTO. The *J* − *V*, EIS, TPV, TPC, and SCLC measures are all carried out with an electrochemical workstation (CHI660E, Chenhua).

## Results and Discussion

The morphology and crystallinity of synthesized pristine MXene materials are verified by SEM, TEM, AFM, and XRD analysis. As shown in SEM image in Fig. S1a, the MXenes show a stacked lamellar structure in cross-sectional view. In the TEM images in Fig. S1b, c, it is clear that the dispersive lamellar structures of MXenes are 2D sheet-like. Then, a representative MXene sheet is measured with AFM. As shown in Fig. S1d and its inset, it is observed that the thickness of 2D MXene sheet is about 2.2 nm, which is close to the 1.5 nm thickness of monolayer MXene in our previous work [31]. As shown in XRD measure in Fig. S2, the main phases at 7.3°, 14.7°, 27.7°, and 60.6° apiece correspond to the (002), (004), (008), and (110) facets, indicating the Ti and Al crystalline phases in MXene [44, 45]. Here, the weak peaks at 36.0° and 41.6° correspond to the impurity of TiC, as a residue in MAX phase precursor [32]. These reveal that the synthesized MXene is in lamellar morphology and typical crystallinity.

**Fig. 2:**
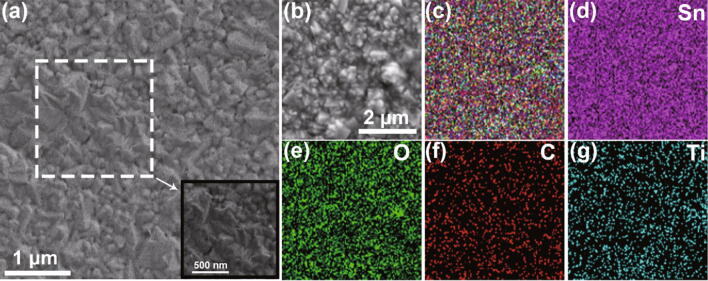
**a** SEM image of MDCN film in top view, with enlarged image of dotted box region in inset. **b** SEM image of MDCN film in top view for EDS analysis, and **c** corresponding element layered image, with elemental mapping image of **d** Sn, **e** O, **f** C, and **g** Ti

With SEM measure, the surface morphology of MDCN film is researched, as shown in Fig. 2a. Note that small flakes sparsely cling on the surface, as indicated in the dotted box. Its chiffon-like structure is clearly shown in the enlarged image in the inset. With the targeted region shown in Fig. 2b, the component of MDCN sample is analyzed in EDS. The elemental layered image and the elemental mapping images for Sn, O, C, and Ti are shown in Fig. 2c–g, respectively. These uniform elemental distributions can be attributed to the SnO_2_ particles effectively spacing the MXene sheets [42] and indicate that the uniform Ti and C doping from MXene precursor.

To investigate the influence from controlled anneal method in Air&N_2_ atmospheres on the crystallinity of MDCN, the test samples of MDCN before and after controlled anneal are fabricated and measured by XRD analysis. In order to achieve more obvious crystalline signals, the samples are fabricated with thick MDCN layers, by repeatedly spin-coating the precursor about 20 times on FTO substrates. As shown in Fig. S3, on the MDCN before controlled anneal, the patterns at 2θ of 26.58°, 33.77°, 37.77°, 51.76°, 61.75°, and 65.74° confirm to the (110), (101), (200), (211), (310), and (301) planes of SnO_2_ crystal in JCPDF No. 46-1088. This indicates the resultant material is mainly SnO_2_ crystal. In addition, compared to this MDCN before controlled anneal, the MDCN after controlled anneal shows the extra patterns at 2θ of 25.28°, 36.95°, 37.80°, 38.58°, 48.05°, 53.89°, 55.06°, 62.89°, and 68.76°, confirming to (101), (103), (004), (112), (200), (105), (211), (204), and (116) planes of anatase TiO_2_ in JCPDF No. 21-1272. These evidence that the anatase TiO_2_ crystals are successfully obtained with this controlled anneal method.

**Fig. 3:**
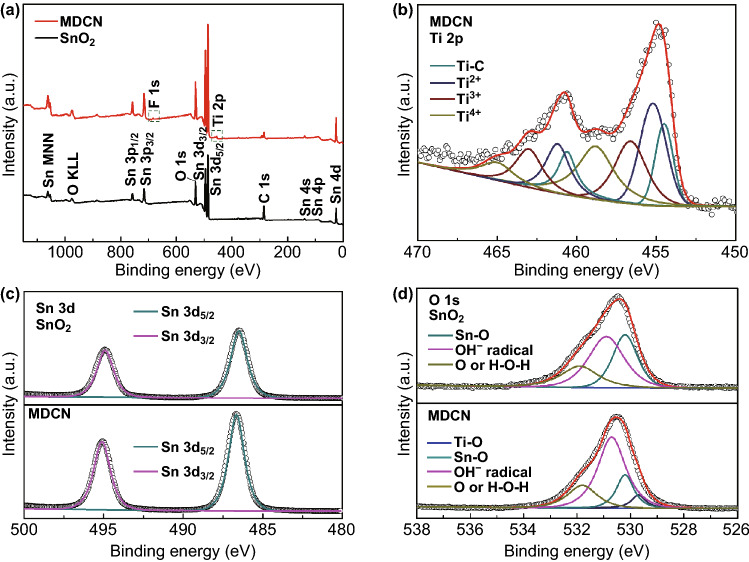
**a** XPS survey spectra of SnO_2_ and MDCN samples. The high-resolution XPS spectra of **b** Ti 2*p* for MDCN sample, **c** Sn 3*d* for SnO_2_, and MDCN samples, and **d** O 1*s* for SnO_2_ and MDCN samples

In order to investigate the valence state transform of material in MDCN compared to pristine SnO_2_ counterpart, the XPS measures for individual element are employed. The survey, Ti 2*p*, Sn 3*d*, and O1*s* spectra are shown in Fig. 3a–d, respectively, where the peak positions have been calibrated by the surface carbon signal at 284.6 eV as an internal standard. In survey spectra in Fig. 3a, it is observed that the Sn, O, and C patterns coexist in pristine SnO_2_ sample. In contrast, extra Ti 2*p* and F 1*s* peaks are noticed for MDCN sample. In addition, the atomic contents of Sn, O, C, Ti, and F have also been obtained as 17.44%, 48.09%, 29.79%, 2.99%, and 1.68%, respectively, by this XPS measure. These indicate that the Ti element in small quantity have been successfully introduced by MXene addition, conforming to the above EDS result. Here, the extra F element can be attributed to the residual LiF corrosion. However, the F elements have not been detected in the above EDS analysis, which may be attributed to its relative tiny amount. Then, the effect from the controlled anneal method on the F content in the MDCN sample is investigated. The MDCN samples before controlled anneal are fabricated with the thickness as same as that for MDCN samples after controlled anneal (i.e., aforementioned MDCN samples). The survey spectra of MDCN before controlled anneal are shown in Fig. S4a, and the extracted F 1*s* spectra of MDCN samples before and after controlled anneal are exhibited in Fig. S4b. In MDCN sample before controlled anneal, three peaks can be disintegrated at 686.8, 685.5, and 684.4 eV, corresponding to F–C, F–Al, and F–Ti bonds, separately. Compared to the MDCN sample before controlled anneal, it is observed that the position and intensity of these three disintegrated peaks for the MDCN sample after controlled anneal is unchanged, indicating the negligible change of F element by this controlled anneal method.

Further, in Ti 2*p* spectra in Fig. 3b, the MDCN sample shows the coexistence of TiC, Ti^2+^, Ti^3+^, and Ti^4+^ bonds, referring to literatures [35, 46, 47]. In order to investigate the Ti change in the MDCN sample, the XPS spectra of pure MXene are measured, with the survey spectrum shown in Fig. S5a and high-resolution Ti 2*p* spectrum shown in Fig. S5b. Note that the pure MXene also shows the same Ti-based bonds as those for MDCN sample. The atomic contents of these different Ti-based bonds in these Ti 2*p* spectra for the two samples are compared in Table S1. It is observed that the Ti^4+^ content in MDCN is obviously larger than that in pure MXene, revealing the Ti elements in other bonds, such as Ti^2+^, Ti^3+^ bonds, in pure MXene have been converted into the Ti^4+^ bond in MDCN. These further demonstrate the existence of TiO_2_ crystal in MDCN, confirming to the above XRD results.

In Sn 3*d* spectra in Fig. 3c, the same peaks at 486.6 and 495.1 eV can be observed in SnO_2_ and MDCN samples, which can be attributed to Sn 3*d*_5/2_ and Sn 3*d*_3/2_, respectively, confirming the successful formation of SnO_2_ phase [45]. In O 1*s* spectra in Fig. 3d, the pristine SnO_2_ sample shows three peaks with binding energies of 530.2, 530.8, and 532.0 eV, which can be attributable to Sn–O, OH^−^ radical, and adsorbed oxygen or adsorbed H_2_O, separately [46, 48, 49]. In contrast, an extra peak is observed for MDCN sample at 529.7 eV, which can be originated from the Ti–O bond [49]. This Ti–O bond can be attributed to the Ti–OH functional group or the Ti-based oxides. Considering the abundant Ti-based bonds of Ti^2+^, Ti^3+^, and Ti^4+^ in the above Ti 2*p* spectrum for MDCN sample, it can be deducted the existence of Ti-based oxides with the Ti–O bond. Therefore, this coexistence of Sn-based oxides with less Ti-based oxides in MDCN sample is beneficial to form a semiconductor oxide heterojunction.

**Fig. 4:**
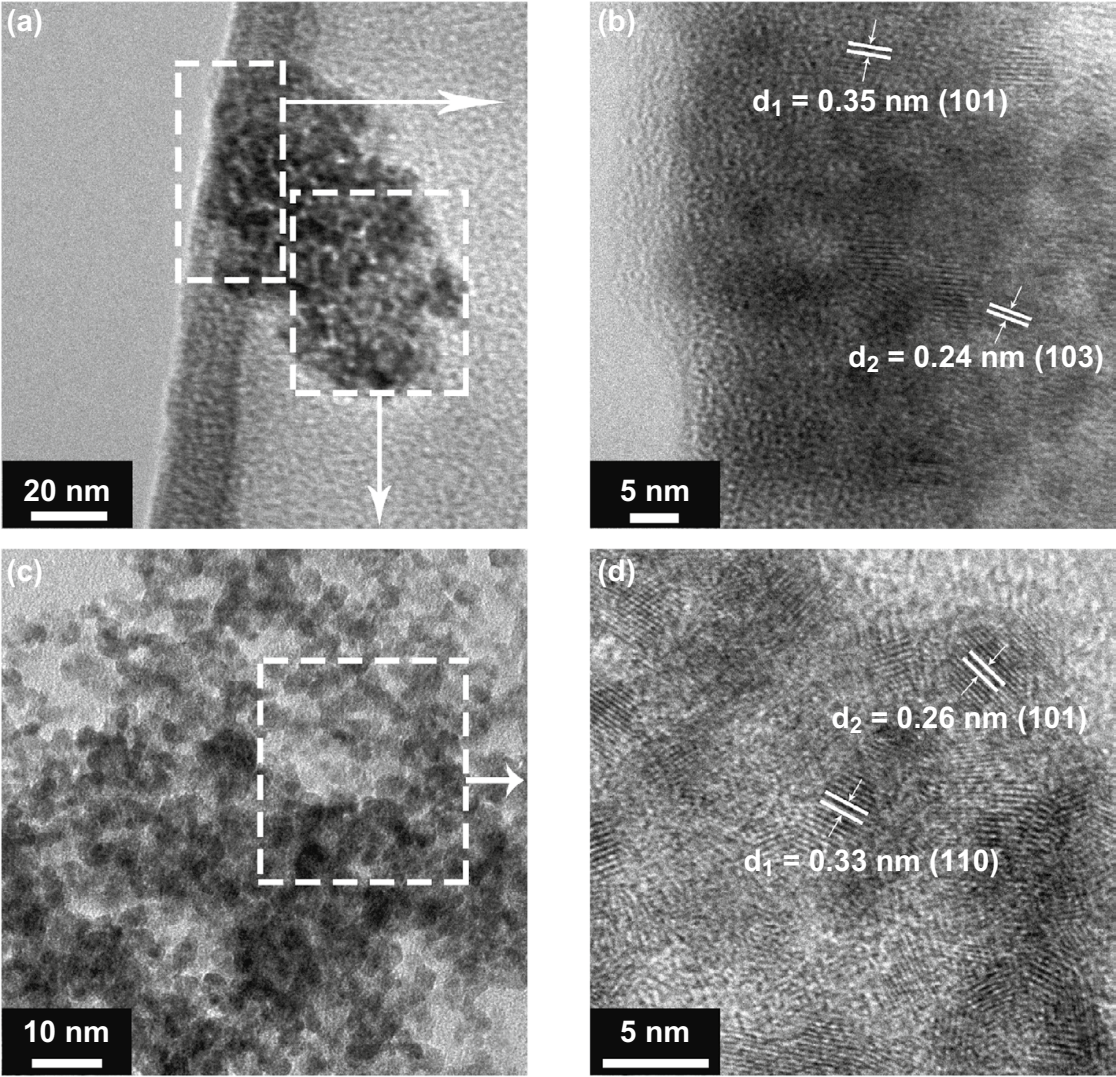
**a** TEM image of MDCN sample. **b** HRTEM image for the magnified left dotted box in **a**. **c** TEM image for the magnified right dotted box in **a**. **d** HRTEM image for the enlarged region in dotted box in **c**

The lattice configurations of MDCN material are further characterized by TEM and HRTEM measures, as shown in Fig. 4. In Fig. 4a, the sample shows a 3D stacked structure. The characteristic regions in left dotted and right dotted boxes in Fig. 4a are enlarged and shown in Fig. 4b, c, respectively. In Fig. 4b, the crystal particles are as small as about 5 nm, inferring the QD phase, and the lattice fringes spacing at *d*_1_ = 0.35 nm and *d*_2_ = 0.24 nm correspond to (101) and (103) planes of anatase TiO_2_, respectively, indicating the 0D anatase TiO_2_ QDs [50]. Therefore, it can be demonstrated that the aforementioned Ti-based oxides in XPS measure is the anatase TiO_2_. In Fig. 4c, abundant nanoscale particles are observed. With its enlarged HRTEM analysis in Fig. 4d, the lattice image spacing of *d*_1_ = 0.33 and *d*_2_ = 0.26 nm corresponds to the (110) and (101) planes of rutile SnO_2_ phases [51], indicating that those stacked 3D structures in Fig. 4a are constructed by SnO_2_ crystals. Therefore, it is evident the intertwining of tiny anatase 0D TiO_2_ QDs and 3D SnO_2_ particles, constructing TiO_2_/SnO_2_ heterojunction structure in the MDCN sample.

**Fig. 5:**
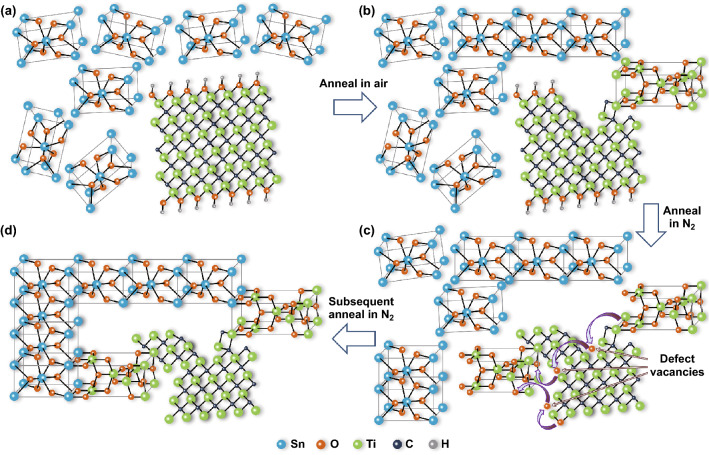
**a**–**d** Generation mechanism of MDCN material

On the basis of the above characterizations, the growth mechanism for MDCN is deduced with atomic models sketched in Fig. 5, where the 2D structure of MXene refers literatures [52]. When the MXene is added into the SnO_2_ precursor, the 2D MXene are dispersed due to strong van der Waals interaction between adjacent nanosheets. The OH^−^ functional group is generated surrounding 2D MXene sheets in the solution, and the SnO_2_ is dispersed around the MXene sheets, as shown in Fig. 5a. In the first anneal procedure in air atmosphere, the rutile SnO_2_ crystal enlarges, and the anatase TiO_2_ crystal is generated by oxidation effect at the edge of MXene sheet, and then the MXene sheet is torn apart to form defect vacancies at the damaged sizes, as shown in Fig. 5b. In the next anneal procedure in N_2_ atmosphere, the SnO_2_ and anatase TiO_2_ crystals are keeping on grow. However, the generation of new TiO_2_ crystal is restricted without outside O element source in N_2_ atmosphere. This requisite O element source can only come from the as-formed TiO_2_ crystal and OH^−^ functional groups. Therefore, in order to affiliate more stable phase in SnO_2_ surroundings, these O elements in as-formed TiO_2_ crystal and OH^−^ functional groups transfer into new-formed anatase TiO_2_ crystal, through jumping at the defect sites in MXene sheet, as shown in Fig. 5c, forming the incomplete crystals for all anatase TiO_2_ because of general lack of O elements. In the subsequent anneal in N_2_, these incomplete crystals tend to assemble with adjacent SnO_2_ crystals to form more stable phase, and then nanoscale TiO_2_/SnO_2_ heterojunctions are formed. Here, the two anneal procedures in N_2_ atmosphere shown in Fig. 5b, c are belonged to the beginning and subsequent procedures of N_2_ anneal for 25 min in a glove box described in the above experimental part. Briefly, the nanoscale anatase TiO_2_ QDs in 0D, derived from the edge region in 2D MXene sheets, are grown and rooted on surrounding SnO_2_ particles in 3D, forming nanoscale TiO_2_/SnO_2_ heterojunctions. Herein, the 2D MXene sheets act as conductive bridges bonding these TiO_2_/SnO_2_ heterojunctions at edge region of sheet, constructing the final MDCN structure, as shown in Fig. 5d.

The wetting property of ETLs is characterized by comparing the contact angles formed by deionized water droplet. As shown in Fig. S6, the contact angle of MDCN ETL is measured only 20°, which is much smaller than that on SnO_2_ ETL (40°), indicating a less hydrophobic surface. This high hydrophilicity offers higher spreading property with polar solvents (such as DMF) on MDCN layer, and then is beneficial to form homogeneous perovskite films. Further, the 3D surface profiles of SnO_2_ and MDCN layer on FTO substrates are measured by AFM, as shown in Fig. S7a, b, respectively. The highly dense uniform surfaces can be observed for these two samples. The root-mean-square values of roughness for SnO_2_ and MDCN samples are obtained about 31.66 and 38.83 nm, suggesting the slight rougher surface construction of MDCN film. Therefore, it is deduced that the higher hydrophilicity of MDCN ETL is mainly affected by the extra MXene and TiO_2_ materials on the surface, which enlarge the surface roughness, and then magnifies the hydrophilicity of SnO_2_ material.

The influence of MXene content in ETL on the crystal size of upper perovskite layer is investigated. The ETLs are fabricated by mixing different MXenes in the SnO_2_ solution. With 0.01, 0.02, 0.03, 0.05, 0.1 wt% MXene additions, the samples are recorded as MDCN-0.01, MDCN-0.02 (i.e., aforementioned MDCN sample), MDCN-0.03, MDCN-0.05, and MDCN-0.1, respectively. The described samples are fabricated in a perovskite/ETL/FTO structure. With pristine SnO_2_, MDCN-0.01, MDCN-0.02, MDCN-0.03, MDCN-0.05, and MDCN-0.1 ETLs, the surface SEM images of resulted perovskite layers in top view are shown in Fig. S8a–f, respectively, and corresponding statistic grain size distributions are drawn in Fig. S8g–l, respectively, and their average grain size is obtained as 452, 525, 658, 485, 463, and 437 nm, respectively. Note that the average grain size increases and then decreases with increasing MXene content, and it achieves the maximum with the MDCN-0.02 ETL. Compared to SnO_2_ sample, the bigger average grain size of perovskite layer on MDCN-0.02 ETL can be attributed to the higher hydrophilicity and the bigger roughness of the below ETL, because of the extra MXene and TiO_2_ materials on the ETL surface as discussed in Figs. S6 and S7. Then, the increase in average grain size of perovskite layer from SnO_2_ to MDCN-0.02 sample can be attributed to the increase in introduced MXene and TiO_2_ materials on the ETL surface, which can roughen the ETL surface. In addition, the decrease in average grain size of perovskite layer from MDCN-0.02 to MDCN-0.1 sample can be attributed to the excessive MXene and TiO_2_ materials on the ETL surface, which will block with each other and then smooth the ETL surface. Further, based on pristine SnO_2_ ETL without MXene content, the perovskite layer shows obvious pin hole, signed with a black dotted circle in Fig. S8a. In contrast, based on the MDCN-0.01 ~ MDCN-0.1 samples, the perovskite layers show compact surfaces almost without pin hole, which can be attributed to the abundant functional groups offered by the extra MXene materials. Here, the MDCN-0.02-based sample, achieving the maximum of average grain size for perovskite layer, is chosen for research in the following sections.

**Fig. 6:**
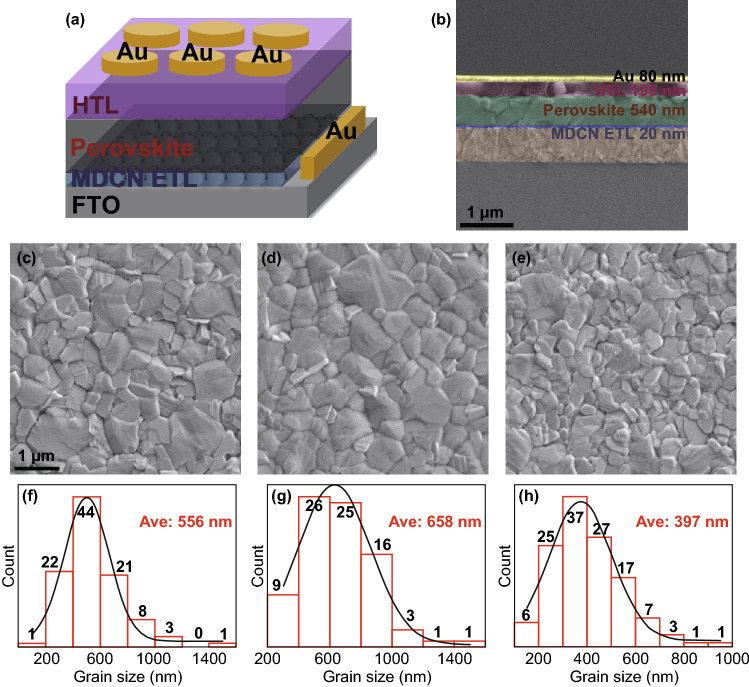
**a** Device structure of a PSC using MDCN as ETL. **b** Cross-sectional SEM image of a complete device with MDCN-0.02 ETL. The SEM images of perovskite layers on the ETL of **c** MDCN-Air, **d** MDCN-Air&N_2_, **e** MDCN-N_2_. **F**–**h** Statistic grain size distributions for perovskite film in **c**–**e**, respectively

The device structure of entire PSC is represented in Fig. 6a, where the functional layers, such as FTO front contact, MDCN ETL, perovskite absorption layer, spiro-OMeTAD HTL, and Au counter electrode, are clearly signed. The SEM image in cross-sectional view for a real device with MDCN-0.02 ETL is shown in Fig. 6b, where the thicknesses of ETL, perovskite layer, HTL, and Au layer are measured as 20, 540, 195, and 80 nm, respectively.

In order to inspect the influence from anneal method of MDCN ETL on the upper perovskite layer, the surface morphology and grain size of resulted perovskite layers are investigated. The samples are also fabricated with a perovskite/ETL/FTO architecture just with the same MDCN-0.02 ETL. The samples annealed with controlled method is recorded as MDCN-Air&N_2_ (i.e., aforementioned MDCN-0.02 or MDCN sample), for comparison, those annealed just in air for 30 min are recorded as MDCN-Air, and those just annealed in N_2_ for 30 min are recorded as MDCN-N_2_. The SEM images of perovskite layers in top view for MDCN-Air, MDCN-Air&N_2_, and MDCN-N_2_ are shown in Fig. 6c–e, respectively, and the corresponding statistic grain size distributions are diagramed in Fig. 6f–h, respectively, and the average grain sizes are obtained as 556, 658, and 397 nm, respectively. Here, Fig. 6d, g is the same as Fig. S8c, i, respectively, for the completely same samples. Note that the MDCN-Air&N_2_ still achieves the maximum average grain size, which can be attributed to the rough surface of below ETL, as discussed in Fig. S8. For the MDCN-Air sample, the smaller average grain size can be attributed to the excessive TiO_2_ on the surface because of sufficient oxidation in air atmosphere, which will passivate and smooth the ETL surface, and then shrinks its hydrophilicity. In addition, for the MDCN-N_2_ sample, the smaller average grain size can be attributed to the lacking TiO_2_ on the surface, on which the 2D MXene sheet can smooth the surface, and then shrinks its hydrophilicity.

**Fig. 7:**
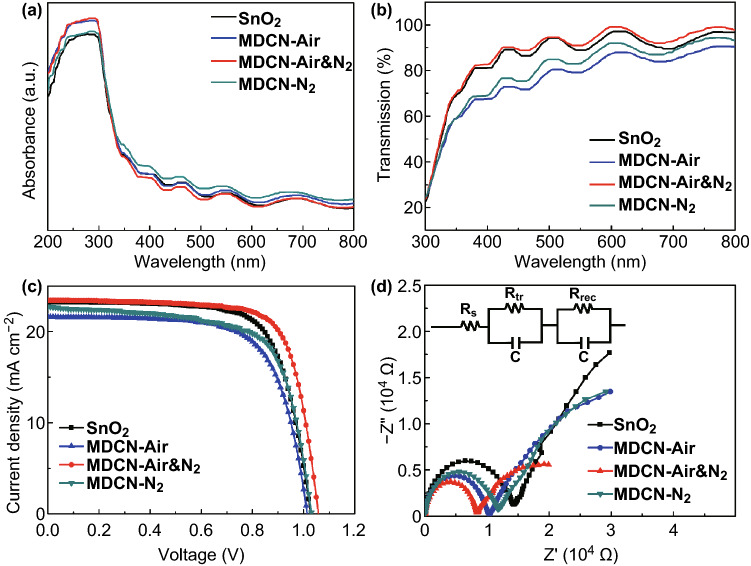
**a** UV–Vis absorption spectra and **b** transmission spectra for SnO_2_, MDCN-Air, MDCN-Air&N_2_, and MDCN-N_2_ ETLs. **c** *J* − *V* curves and **d** Nyquist plots of EIS for PSCs using SnO_2_, MDCN-Air, MDCN-Air&N_2_, and MDCN-N_2_ ETLs. The equivalent circuit of **d** shown in the inset

The optical features of ETL are important factors to affect the PSC performance. With different MXene contents, the transmission spectra of SnO_2_, MDCN-0.01, MDCN-0.02, MDCN-0.03, MDCN-0.05, and MDCN-0.1 ETLs are expressed in Fig. S9. It is observed that the MDCN-0.02 ETL shows a better transmittance feature. Further, with different anneal methods, the absorption and transmission spectra of ETLs are further investigated, as shown in Fig. 7a, b, respectively, for the SnO_2_, MDCN-Air, MDCN-Air&N_2_ and MDCN-N_2_ ETLs. Here, these absorption or transmission spectra are slightly wavy. It is because that a higher power of incident light is set in test for distinguishing the slight differences of samples, by which the irregular background signals are also magnified. However, in the same test conditions, it is not affected to analyze the intensity difference of test samples. In Fig. 7a, in the UV light region ranging from 200 to 380 nm, the light absorption intensities of MDCN-Air&N_2_ and MDCN-Air are approximate, and they are larger than those of SnO_2_ sample, confirming the inactive UV photocatalysis of SnO_2_ material [13]. In the visible light region ranging from 380 to 780 nm wavelength, the light absorption and transmission properties of these samples are different, which can be attributed to the introduced MXene material and the resultant film nanostructure. When the MXene is introduced into the SnO_2_ materials, on the one hand, the MXenes as barriers in MDCN can increase the light absorption and then decrease the light transmission. On the other hand, the relatively rough surface of MDCN sample in comparison to SnO_2_ sample, as discussed in the above AFM measured in Fig. S7, can reduce the light reflection and then enlarge the light transmission, based on light scattering effect in its suede-like rough surface. Then, it can be concluded that the MXene dopants offer a harmonious optical influence composed with two interinhibitive effects of light-barrier and anti-reflection. Here, compared to SnO_2_ sample, the MDCN-N_2_ sample shows the enhanced absorption and declined transmission. This can be attributed to the dominant light-barrier effect of MXene dopant. Then, compared to this MDCN-N_2_ sample, the MDCN-Air sample shows the declined absorption and declined transmission. This can be attributed to the extra introduced excessive TiO_2_ additions, which concentrate near the surface of SnO_2_ layer because of oxidation from surrounding air, as discussed in aforementioned section. Generally, the TiO_2_ as extra light-barrier will further enhance absorption and decline transmission. However, the declined absorption here can be ascribed to the higher reflection on surface, because of the excessive TiO_2_ additions can passivate the antireflection effect of MXene. In addition, compared to the MDCN-Air sample or even the SnO_2_ sample, the MDCN-Air&N_2_ sample shows the declined absorption and enhanced transmission, which can be ascribed to the dominant antireflection effect of MXene dopants. Therefore, integrating the above results, it can be observed that the light absorption for perovskite layer will not be decreased by the MDCN-Air&N_2_ ETL, because of its superior optical absorption and transmittance properties.

The *J* − *V* performance of PSCs based on MDCN ETLs with different MXene contents is investigated. The *J* − *V* curves of PSCs with SnO_2_, MDCN-0.01, MDCN-0.02, MDCN-0.03, MDCN-0.05, and MDCN-0.1 ETLs are shown in Fig. S10a, and the photovoltaic parameters, such as open-circuit voltage (*V*_OC_), short current density (*J*_SC_), filling factor (FF) and PCE, are listed in Table S2. In general, the *V*_OC_, *J*_SC_, FF, and PCE values all increase and then decrease with increasing MXene content. Here, the MDCN-0.02 ETL-based PSC achieves the highest average PCE of 18.44%. In contrast, the SnO_2_-based counterpart only exhibits an average PCE of 16.42%. These devices are further investigated by EIS measure, with Nyquist plots shown in Fig. S10 b with equivalent circuit in inset. The series resistance (*R*_s_) mainly reflects electrical contacts, wires, and sheet resistance of the electrodes [43, 53]. The charge-transfer resistance (*R*_tr_) means the charge-transfer property at the interface, and *R*_rec_ means the recombination of carriers [43, 54]. Fitted with ZView software, it can be observed that the *R*_s_ values are similar for these samples. With increasing MXene content, the *R*_tr_ value decreases and then increases, revealing that the charge-transfer speed at perovskite/ETL interface increases and then decreases, and the minimum *R*_tr_ value of MDCN-0.02-based sample means the fastest charge transfer, which can be used to explain the almost same change regularity of FF for corresponding devices, as listed in Table S2.

The *J* − *V* performance of PSCs based on MDCN-0.02 ETL with different anneal methods is further investigated. The typical *J* − *V* curves are shown in Fig. 7c with photovoltaic parameters listed in Table 1. The *J* − *V* curve and photovoltaic parameters for the MDCN-Air&N_2_ sample are the same as those of MDCN-0.02-based sample described in Fig. S10a and Table S2, because of the completely same sample. It is observed that the device based on MDCN-Air&N_2_ ETL still shows the highest PCE than others. As shown in Fig. S11, the histogram of photovoltaic parameters for SnO_2_ and MDCN-Air&N_2_-based PSCs further exhibits the repeatability of high performance of MDCN-Air&N_2_-based devices. The performance is further analyzed by EIS measure, and their Nyquist plots are shown in Fig. 7d with equivalent circuit in inset. Here, the plot for MDCN-Air&N_2_ sample is the same to MDCN-0.02-based sample described in Fig. S10b because of the completely same sample. Among these samples, their R_s_ values are similar, and hence, the main contribution for PCE can be attributed to their different *R*_tr_ values. In addition, it is observed that the FF plays an important role on the PCE, therefore, the difference in FF can be explained by the *R*_tr_ regularity. Here, the charge-transfer speed in device, reversely denoted by the *R*_tr_ value, can come from two types of interfaces. One type is the perovskite/MXene interface between perovskite layer and ETL, and another type is the nanoscale interface of TiO_2_/SnO_2_ heterojunction inside the ETL. Compared to SnO_2_-based sample, the smaller *R*_tr_ value of MDCN-N_2_-based sample can be attributed to the faster charge transfer at perovskite/MXene interface, because TiO_2_/SnO_2_ heterojunctions cannot be formed without air atmosphere. Compared to MDCN-N_2_-based sample, the smaller *R*_tr_ value of MDCN-Air-based sample can be attributed to the faster charge transfer through TiO_2_/SnO_2_ heterojunction, because the sufficient heterojunctions generated by oxidation in air atmosphere near the ETL surface, which offer more contribution between perovskite layer and ETL. Further, compared to MDCN-Air-based sample, the smaller *R*_tr_ value of MDCN-Air&N_2_-based sample can be attributed to the fastest charge transfer through these two types of interfaces, because the optimal MDCN structure generated in these regions. In addition, it is observed that the FF or PCE parameter generally shows the negative relationship with the *R*_tr_ value, indicating that the main influence on FF or PCE is the charge-transfer speed at interfaces. However, the FF or PCE parameter of MDCN-N_2_-based sample is not suitable for this regularity. Compared to SnO_2_-based sample, the FF or PCE parameter of MDCN-N_2_-based sample is smaller, although it owns the faster charge transfer as discussed above. This can be attributed to the defects of MXene inside the ETL, which capture and then recombine the traveling charges, and hence weaken the contribution from the faster charge-transfer speed.

**Table 1:** Device performance of PSCs with SnO_2_, MDCN-Air, MDCN-Air&N_2_, and MDCN-N_2_ ETLs (30 samples for each type of ETL)

ETL	*V*_OC_ (V)	*J*_SC_ (mA cm^−2^)	FF (%)	PCE (%)
SnO_2_	1.03 ± 0.04	22.51 ± 1.56	70.55 ± 3.11	16.42 ± 0.41
MDCN-Air	1.04 ± 0.04	22.85 ± 1.20	72.63 ± 3.04	17.34 ± 0.55
MDCN-Air&N_2_	1.07 ± 0.03	23.13 ± 1.39	74.62 ± 3.35	18.44 ± 0.70
MDCN-N_2_	1.02 ± 0.07	21.86 ± 2.50	68.62 ± 5.07	15.36 ± 0.86

**Fig. 8:**
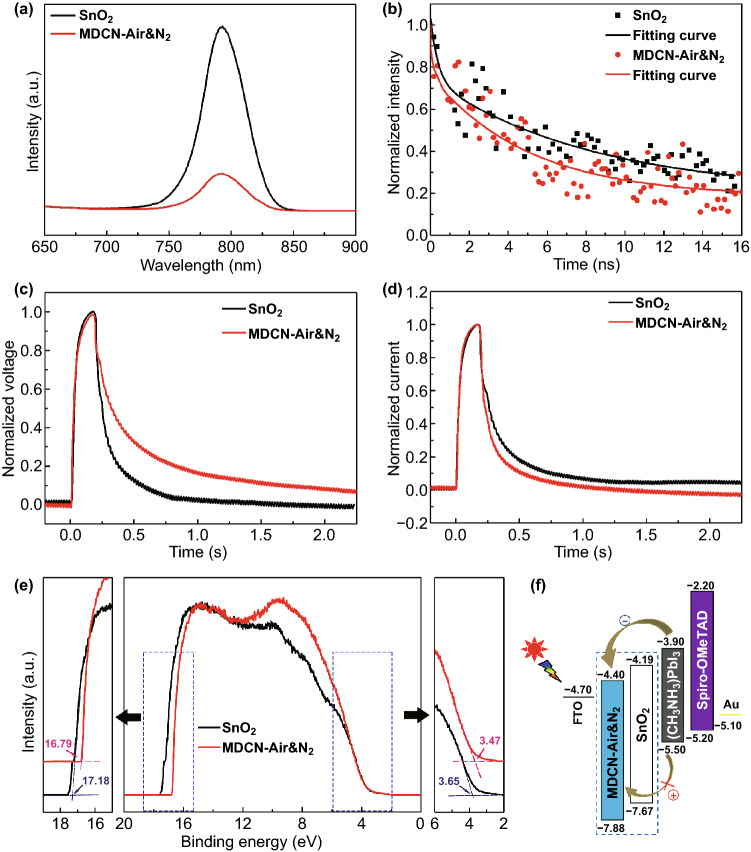
**a** Steady-state PL spectra, **b** TRPL spectra, **c** TPV curves, **d** TPC curves, and **e** UPS spectra for SnO_2_ and MDCN-Air&N_2_ ETLs. **f** Energy diagram for a working device with potential ETLs signed in the dotted box

The steady-state PL spectra for SnO_2_ and MDCN-Air&N_2_ samples are measured and shown in Fig. 8a. The test samples are prepared with a device architecture of perovskite/ETL/FTO with the same experimental procedures described above. Note that these two samples both show strong peaks around 790 nm, and the peak of MDCN-Air&N_2_ is weaker than that of SnO_2_ sample, suggesting stronger electron quenching and transport ability of MDCN-Air&N_2_ ETL [21].

The charge recombination kinetics of ETL is further investigated with the TRPL decay measurement, with a test device architecture as same as that for steady-state PL. The PL decay time, *Y*(*t*), is fitted using a bi-exponential equation (Eq. 1) [43]:

1 $$Y\left( t \right) = A_{1} \exp^{{\left( { - \frac{t}{{\tau_{1} }}} \right)}} + A_{2} \exp^{{\left( { - \frac{t}{{\tau_{2} }}} \right)}} + A_{0}$$

The average PL decay time (*τ*_ave_) is calculated by the *τ*_*i*_ and *A*_*i*_ values of fitted curve data with Eq. 2:

2 $$\tau_{\text{ave}} = \frac{{\sum A_{i} \tau_{i}^{2} }}{{\sum A_{i} \tau_{i} }}\;\;\left( {i = 1, \, 2} \right)$$

The fast decay lifetime (*τ*_1_) is attributed to the trap-assisted recombination by quenching of photo-generated free carriers transported from the perovskite layer to the ETL, and the slow decay lifetime (*τ*_2_) originates from the free carrier recombination before charge collection [55, 56]. With the SnO_2_ and MDCN-Air&N_2_ ETLs, the TRPL spectra are shown in Fig. 8b and detailed fitting parameters are listed in Table 2. Compared to SnO_2_ sample, the MDCN-Air&N_2_ sample shows the similar *τ*_1_ value, and shortened *τ*_2_ and *τ*_ave_ values. The shortened *τ*_2_ and *τ*_ave_ values demonstrate more efficient electron extraction at the perovskite/MDCN-Air&N_2_ interface [55, 57]. This can be attributed to reduction of defect density and formation of good interface at perovskite/MDCN-Air&N_2_ interface. These results are still in agreement with the above steady-state PL results.

**Table 2:** The fitted results of TRPL for various ETLs

ETL	*τ*_1_ (ns)	*τ*_2_ (ns)	*A*_1_	*A*_2_	*τ*_ave_ (ns)
SnO_2_	0.38	8.34	0.29	0.54	8.15
MDCN-Air&N_2_	0.41	5.04	0.28	0.67	5.05

Further, the TPV and TPC measures are conducted to study the charge recombination and extraction properties of devices. The TPV is used to measure charge recombination under the V_OC_ condition, and the decaying progress of V_OC_ means that the recombination occurring of charges. As plotted in Fig. 8c in TPV curve, the *V*_OC_ for MDCN-Air&N_2_-based device shows the slower decay, compared that for SnO_2_-based device, suggesting that the reduced charge recombination occurring within the device based on MDCN-Air&N_2_ ETL [58]. As plotted in Fig. 8d in TPC curve, the short-circuit current for MDCN-Air&N_2_-based device shows the faster decay, compared to that for SnO_2_-based device, implying the enhanced charge transfer, reduced interfacial defects, and better crystal of perovskite layer in MDCN-Air&N_2_-based device [58].

The electron mobility (*μ*) is an important parameter to characterize semiconductive properties [59–61]. It can be obtained by SCLC measure with Eq. 3:

3 $$J = \frac{9}{8}\mu \varepsilon_{0} \varepsilon_{r} \frac{{V^{2} }}{{d^{3} }}$$

where *J* is the current density, *ε*_0_ the vacuum permittivity of 8.85 × 10^−12^ F m^−1^, *ε*_*r*_ the relative dielectric constant of material (13 for SnO_2_) [62], *V* the applied bias, and *d* the ETL thickness. Then, the SCLC measures of SnO_2_ and MDCN-Air&N_2_-based electron only devices are carried out, with the preparation according to the literature [57], and the resultant curves are shown in Fig. S12. It can be calculated that the *μ* value of SnO_2_ is 8.29 × 10^−5^ cm^2^ V^−1^ s^−1^, conforming to literature reports [57]. In contrast, the MDCN-Air&N_2_ layer achieves the higher *μ* value of 4.06 × 10^−4^ cm^2^ V^−1^ s^−1^, indicating the lower defects in materials.

The energy level of MDCN-Air&N_2_ and SnO_2_ is measured with UPS. The test samples are fabricated by spin-coating the precursors on FTO substrates. As shown in the middle plot in Fig. 8e, the left and right intersection regions relative to baseline in dotted boxes are enlarged, respectively. Then, the cutoff binding energy (*E*_CUT_) or Fermi edge (*E*_D_) can be determined by the left or right intersection of baseline with the tangent line, in the left or right enlarged plots. Then, the energy levels at valence band maximum (*E*_VBM_) and conduction band minimum (*E*_CBM_) can be obtained with *E*_VBM_ = *E*_F_ − *E*_D_ (eV) and *E*_CBM_ = *E*_VBM_ + *E*_G_ (eV). Here, the Fermi level (*E*_F_) can be obtained by *E*_F_ = *E*_CUT_ − *E*_IP_, where the *E*_IP_ = 21.2 eV is the energy of incident photon in this test, and the *E*_G_ value is obtained from the UV–Vis absorption spectrum. As shown in Fig. S13, the absorption onset (*λ*_onset_) values for SnO_2_ and MDCN-Air&N_2_ samples are determined and signed by their absorption edges, based on the UV–Vis absorption spectra in Fig. 7a. It is observed that the *λ*_onset_ values for both samples are the same as 365 nm, deducing their same *E*_G_ values of 3.48 eV, based on the *E*_G_ = 1240/*λ*_onset_. On the basis of the above calculations, the energy-level parameters of *λ*_onset_, *E*_G_, *E*_CBM_, and *E*_VBM_ values are concluded and listed in Table 3. It is observed that the *E*_CBM_ and *E*_VBM_ values both downshift 0.21 eV.

**Table 3:** Summary of the energy-level parameters for various ETLs

ETL	*λ*_onset_ (nm)	*E*_G_ (eV)	*E*_CBM_ (eV)	*E*_VBM_ (eV)
SnO_2_	356	3.48	− 7.67	− 7.88
MDCN-Air&N_2_	356	3.48	− 4.19	− 4.40

The schematic energy-level diagram for each layer in working PSC is plotted in Fig. 8f, based on the above energy-level parameters, in which the SnO_2_ and MDCN-Air&N_2_ are alternative ETLs signed in a dotted box. The energy levels of FTO, (CH_3_NH_3_)PbI_3_, Spiro-OMeTAD refer to our previous work [21], and that of Au is from the literature [63]. In comparison with the SnO_2_ ETL, the downshifted *E*_VBM_ of MDCN-Air&N_2_ ETL leads to the higher ability for suppressing the hole recombination toward the FTO layer and then contributes to the higher PCE, although the downshifted *E*_CBM_ means the stronger ability for extracting electron from perovskite layer. Nevertheless, the downshifted *E*_CBM_ also means more recombination probability for the electrons at the conduction band of ETL, relative to the holes at the valence band of perovskite layer, which will weaken the improvement of PCE of devices.

**Fig. 9:**
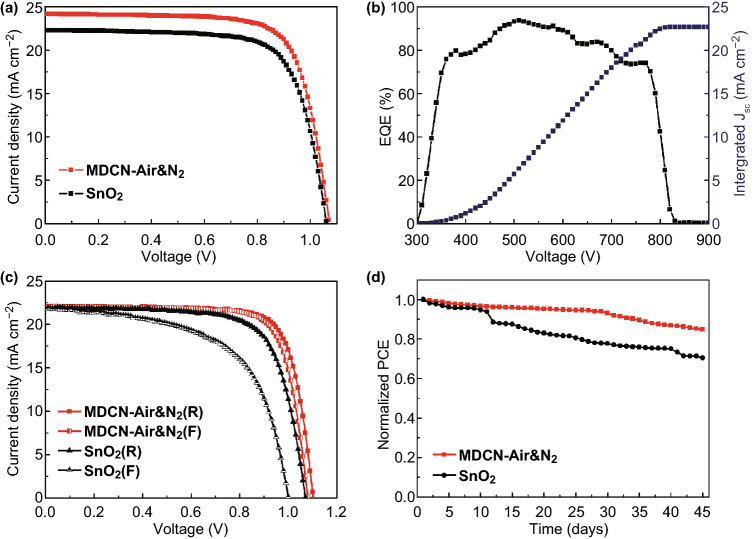
**a** *J* − *V* curves of champion PSCs with MDCN-Air&N_2_ and SnO_2_ ETL. **b** EQE spectrum and corresponding integrated J_SC_ of PSC with MDCN-Air&N_2_ ETL. **c** Hysteresis *J* − *V* curves and **d** PCE stability of PSCs with MDCN-Air&N_2_ and SnO_2_ ETL

The device performances are further demonstrated by investigating their champion PCE. As shown in *J* − *V* curves in Fig. 9a, for MDCN-Air&N_2_-based sample, the champion PCE is 19.14%, with *J*_SC_ of 24.16 mA cm^−2^, *V*_OC_ of 1.07 V and FF of 74.05%. In contrast, the champion PCE for SnO_2_-based sample is only 16.83%, with *J*_SC_ of 21.88 mA cm^−2^, *V*_OC_ of 1.07 V, and FF of 71.90%. The EQE of MDCN-Air&N_2_-based device is measured. As shown in Fig. 9b, the spectrum expresses a wide photo-response from 350 to 800 nm with a peak of ≈ 92% at ≈ 510 nm. The integrated current density from the EQE is obtained as 22.68 mA cm^−2^, which is almost in agreement with the measure from the solar simulator in this research.

In order to correctly evaluate device performance, the hysteresis characteristics of typical cells with SnO_2_ and MDCN-Air&N_2_ ETLs are measured with a single *J* − *V* loop, as shown in Fig. 9c, where the symbol of (R) or (F) indicates the reverse or forward scan method, respectively, with the *V*_OC_, *J*_SC_, FF, and PCE listed in Table 4. The hysteresis index HI = (PCE(R) − PCE(F))/PCE(R) of SnO_2_ and MDCN-Air&N_2_ device is calculated as 0.10 and 0.03. The far less HI of MDCN-Air&N_2_-based device further reveals the electron extraction is effectively accelerated with the MDCN structure.

**Table 4:** Photovoltaic parameters of PSCs with SnO_2_ and MDCN-Air&N_2_ ETLs measured under reverse and forward voltage scanning

ETL	*V*_OC_ (V)	*J*_SC_ (mA cm^−2^)	FF (%)	PCE (%)
SnO_2_(R)	1.07	21.88	71.89	16.83
SnO_2_(F)	1.02	21.73	67.74	15.01
MDCN-Air&N_2_(R)	1.10	22.03	77.78	18.90
MDCN-Air&N_2_(F)	1.08	22.01	77.12	18.33

Moreover, the moisture-resistance ability of PSC is evaluated by testing the devices without encapsulation in 30–40% humidity air at room temperature. As shown in Fig. 9d, the device with MDCN-Air&N_2_ ETL exhibits high stability retaining almost 85% of initial PCE, even after 45 days. In contrast, the device with SnO_2_ ETL degrades below 75% of its initial performance in the same test environment. This represents the superior moisture-resistance ability of the perovskite layer, which is attributed to the unique MDCN structure in below ETL.

## Conclusion

With a controlled anneal method implemented in air and then in N_2_ atmospheres, a film in MDCN structure is successfully designed and fabricated, by means of adopting the Ti_3_C_2_T_*X*_ MXene into SnO_2_ precursor. In the resulted MDCN, the 0D anatase TiO_2_ quantum dots, surrounding on 2D conducting Ti_3_C_2_T_*X*_ sheets, are in situ rooted on 3D SnO_2_ nanoparticles, constructing nanoscale TiO_2_/SnO_2_ heterojunctions, with an oxygen vacancy scramble effect. With the optimized MDCN-0.02 ETL, the optical transmittance of ETL is enhanced, the interface impedance is decreased, and the crystallinity of upper perovskite layer is enlarged, achieving more amount carrier and the rapider carrier transfer. The champion PCE of resulted PSC achieves 19.14%, yet that of counterpart is just 16.83%. It can also maintain almost 85% of its initial performance for more than 45 days in 30–40% humidity air, and the counterpart comparatively degrades below 75% of its initial performance.

## Electronic supplementary material

Below is the link to the electronic supplementary material.

Supplementary material 1 (PDF 1516 kb)
