# Supplementary material for: Low-Temperature Growing Anatase TiO2/SnO2 Multi-dimensional Heterojunctions at MXene Conductive Network for High-Efficient Perovskite Solar Cells
Source: Nanomicro Lett. 2020 Jan 31;12:44. doi: 10.1007/s40820-020-0379-5 (PMC7770768; doi:10.1007/s40820-020-0379-5)
Supplement: Supplementary file 1 — Supplementary material 1 (PDF 1516 kb) [file 40820_2020_379_MOESM1_ESM.pdf]

Supporting Information for

Low-temperature Growing Anatase-TiO_2/SnO_2 Multi-dimensional Heterojunctions at MXene Conductive Network for High-efficient Perovskite Solar Cells

Linsheng Huang[1], Xiaowen Zhou[1], Rui Xue[1], Pengfei Xu[1], Siliang Wang[1], Chao Xu[1], Wei Zeng [1,] * Yi Xiong[2, 3,] *, Hongqian Sang[3, 4], Dong Liang[1]

[1]National Engineering Research Center for Agro-Ecological Big Data Analysis & Application, School of Electronics and Information Engineering, Anhui University, No.111 Jiulong Road, Hefei 230601, People's Republic of China

[2]Science and Technology Institute, Hubei Key Laboratory of Biomass Fibers & Eco-Dyeing & Finishing， Wuhan Textile University, Wuhan 430073, People's Republic of China

[3]School of Physics and Technology, MOE Key Laboratory of Artificial Micro- and Nano-Structures and Center for Electron Microscopy, Wuhan University, Wuhan 430072, People's Republic of China

[4]Department of Physics, King's College London, The Strand, London WC2R 2LS, United Kingdom

*Corresponding authors. E-mail: youfmail@163.com (Wei Zeng); xiong@wtu.edu.cn or xiongyi@whu.edu.cn (Yi Xiong)

Supplementary Figures

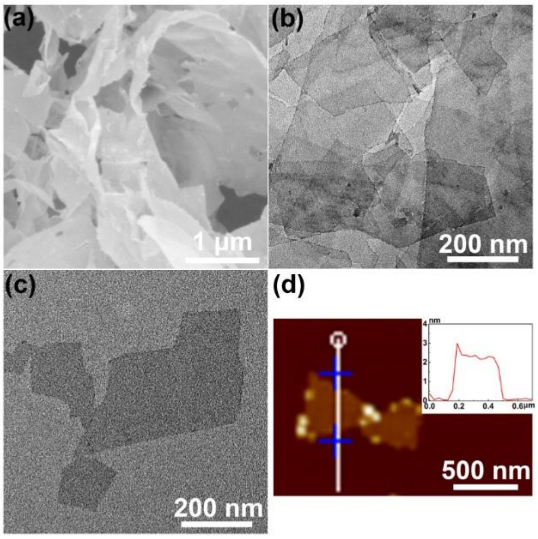

Fig. S1 (**a**) The SEM morphology, (**b-c**) TEM morphologies, and (**d**) AFM surface morphology of resulted MXene material with its height profile in inset

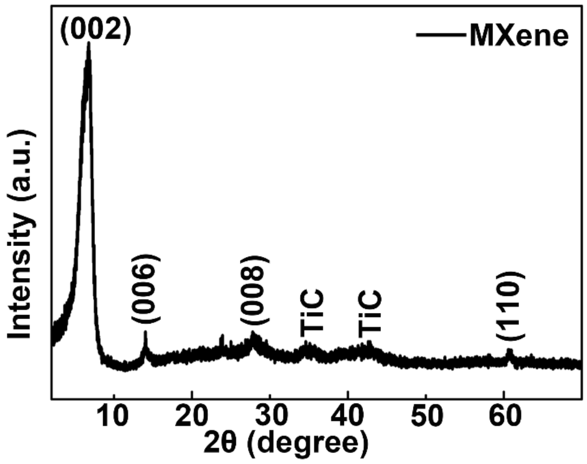

Fig. S2 XRD pattern of resulted MXene material

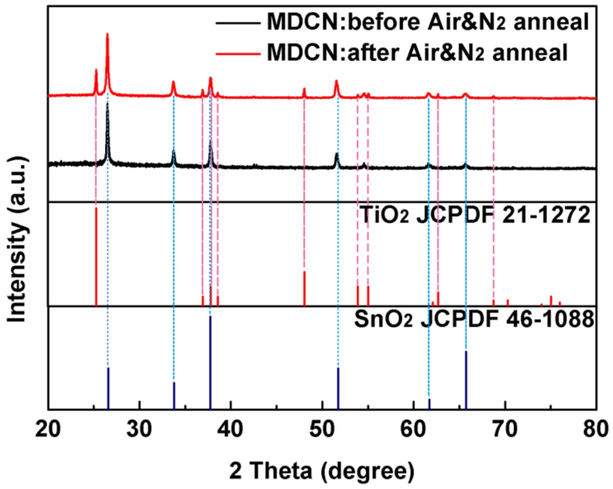

Fig. S3 XRD patterns of MDCN before and after controlled anneal method in Air&N_2 atmospheres

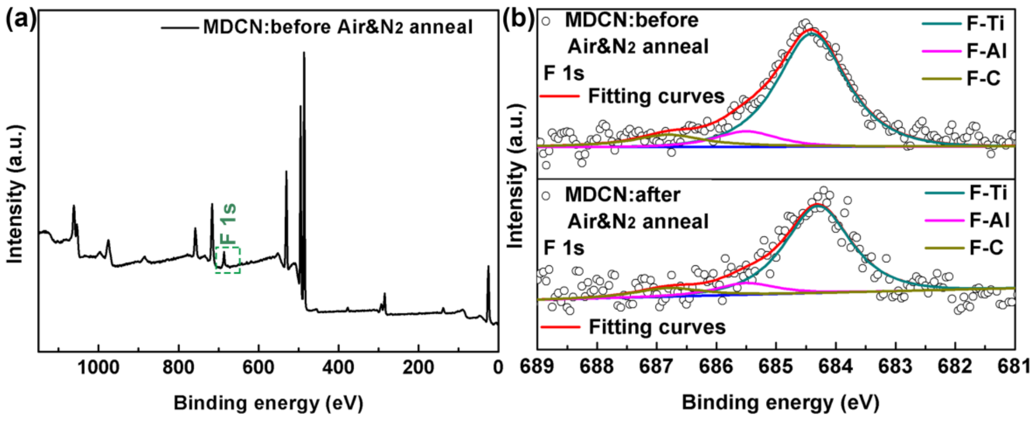

Fig. S4 a The survey spectra of MDCN before controlled anneal. **b** The extracted F 1s spectra of MDCN samples before and after controlled anneal

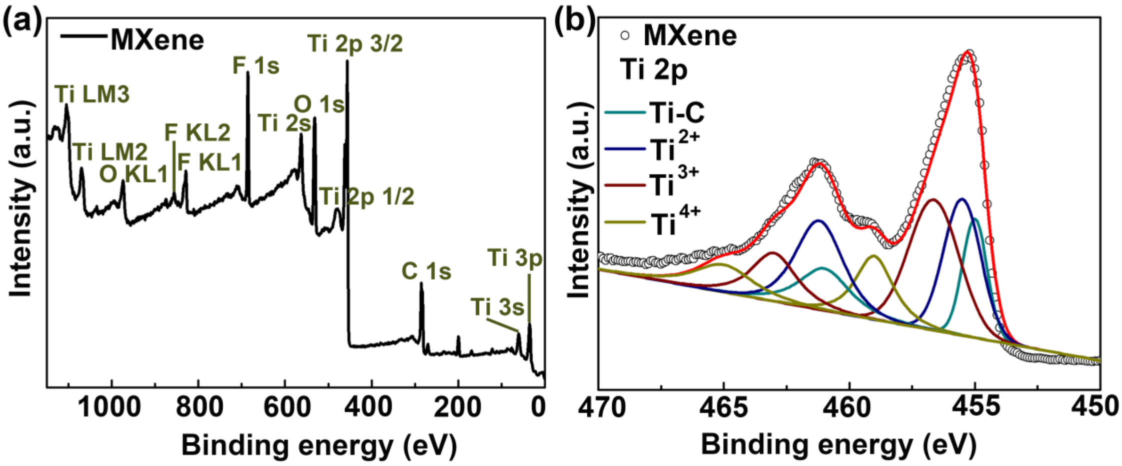

Fig. S5 Survey spectrum of **a** pure MXene **b** with its Ti 2p

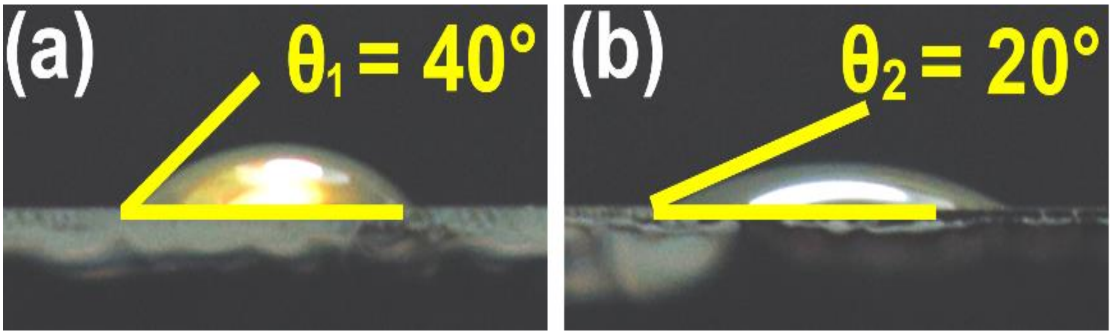

Fig. S6 Wetting angles of a water droplet on the (**a**) SnO_2 and (**b**) MDCN films

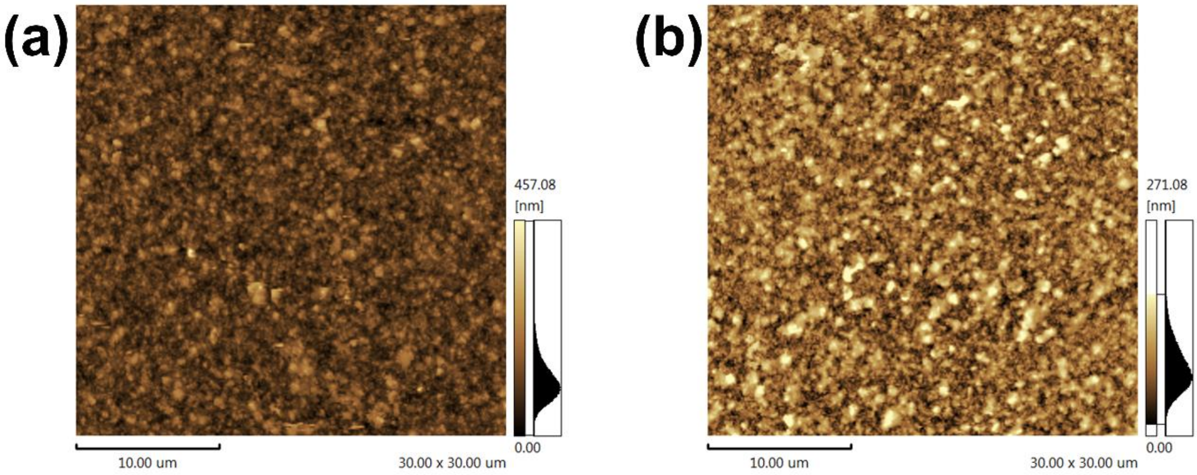

Fig. S7 AFM images of (**a**) SnO_2 and (**b**) MDCN surfaces

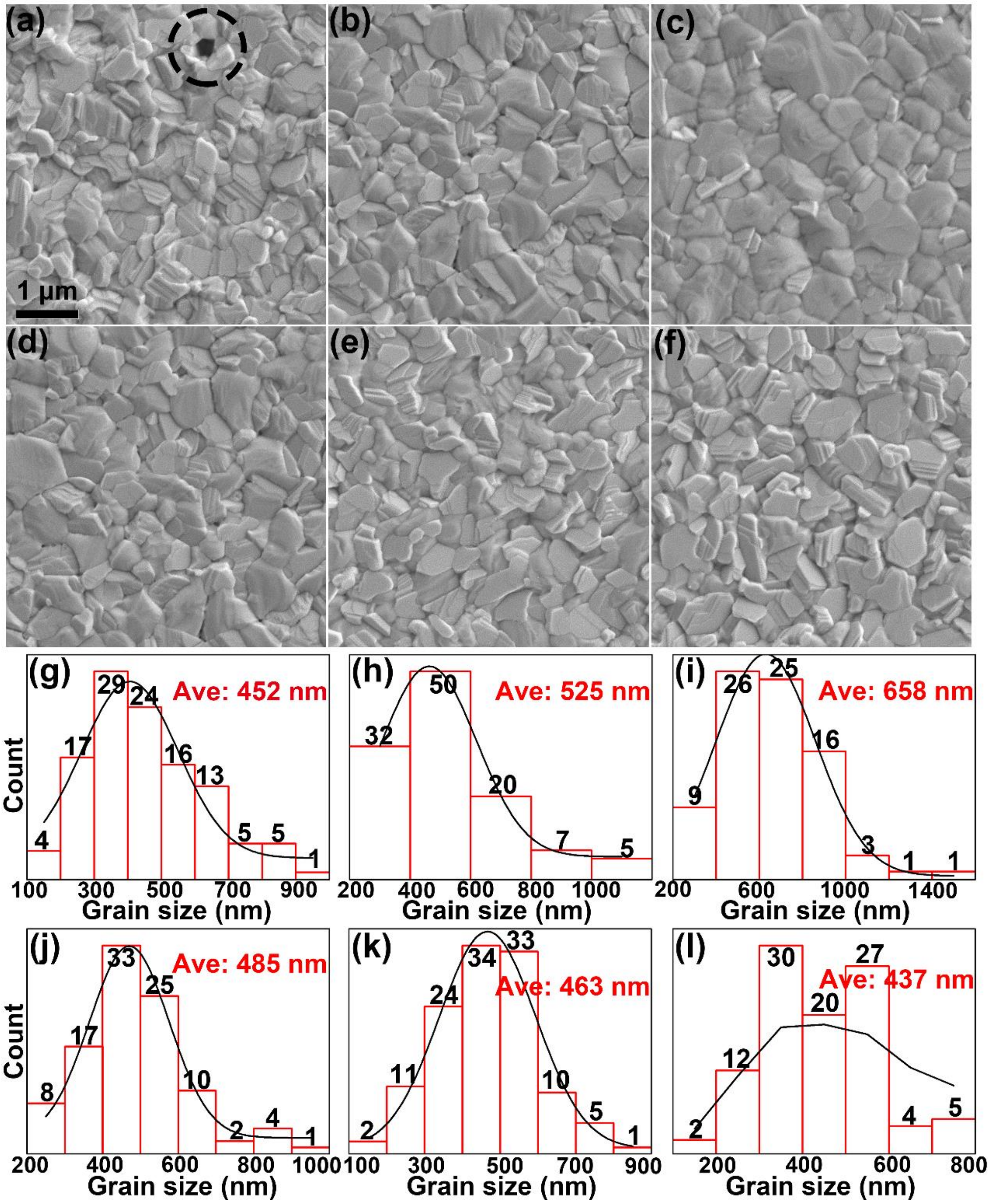

Fig. S8 Top-view SEM images of perovskite/ETL/FTO with different ETL: (**a**) SnO_2; (**b**) MDCN-0.01; (c) MDCN-0.02 (i.e., MDCN sample); (**d**) MDCN-0.03; (**e**) MDCN-0.05; (**f**) MDCN-0.1. (**g-l**) Statistic grain size distributions for perovskite film in (**a-f**)

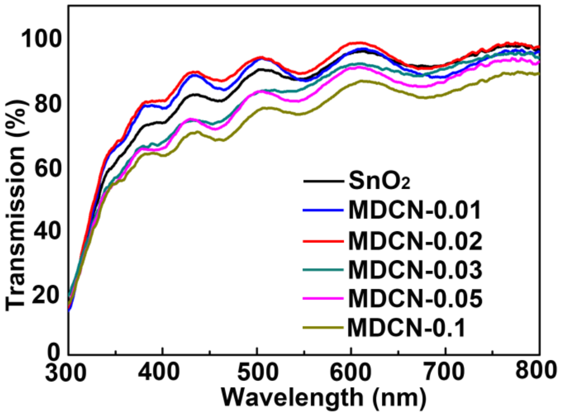

Fig. S9 Transmittance spectra of SnO_2, MDCN-0.01, MDCN-0.02, MDCN-0.03, MDCN-0.05, and MDCN-0.1 ETLs

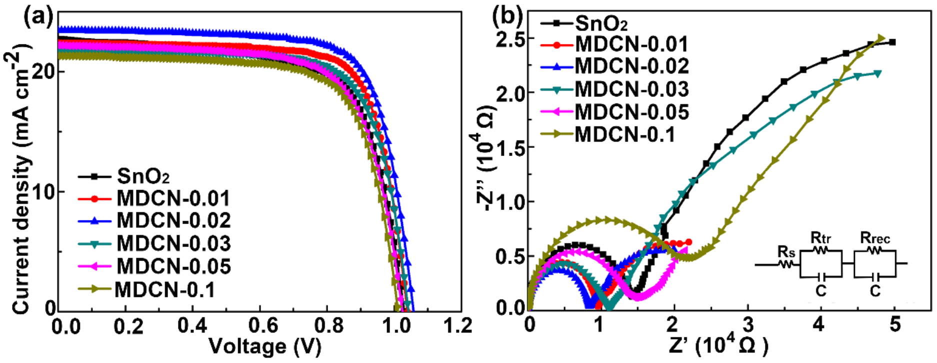

Fig. S10 (**a**) J-V curves and (**b**) Nyquist plots of EIS for PSCs using SnO_2, MDCN-0.01, MDCN-0.02, MDCN-0.03, MDCN-0.05, and MDCN-0.1 as ETLs. The equivalent circuit for (**b**) shown its inset.

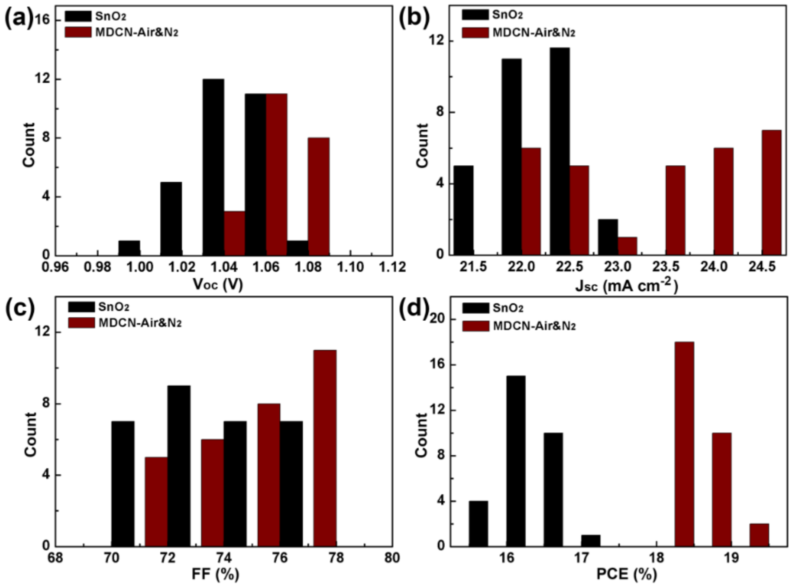

Fig. S11 Histogram of parameters of PSCs with SnO_2 and MDCN-Air&N_2 ETLs

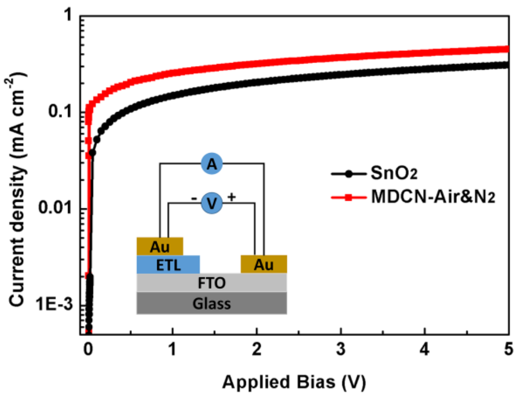

Fig. S12 The SCLC curve of SnO_2 and MDCN-Air&N_2 layers with the test devices sketch in inset

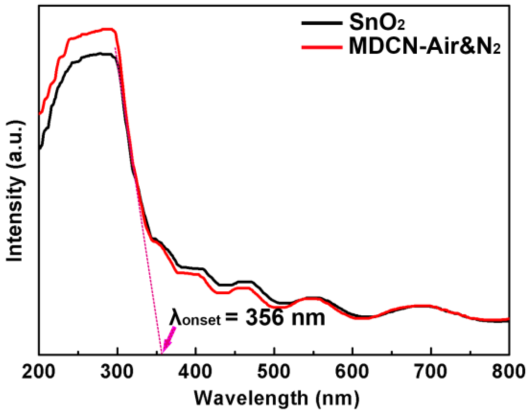

Fig. S13 Absorption onset value measure for SnO_2 and MDCN-Air&N_2 samples with UV-vis absorption spectra

Table S1 Atomic contents of different Ti-based bonds for pure MXene and MDCN

	Ti-C	Ti^{2+}	Ti^{3+}	Ti^{4+}
Pure MXene	18.76	33.91	31.67	15.65
MDCN	19.94	31.27	25.39	23.40

Table S2 Device performance of PSCs based on ETLs with different MXene contents (30 samples for each type of ETL)

ETL	V_{OC} (V)	J_{SC} (mA cm^{-2})	*FF* (%)	PCE (%)
SnO_2	1.03 ± 0.04	22.51 ± 1.56	70.55 ± 3.11	16.42 ± 0.41
MDCN-0.01	1.05 ± 0.05	22.73 ± 1.65	72.89 ± 3.50	17.38 ± 0.48
MDCN-0.02	1.07 ± 0.03	23.13 ± 1.39	74.62 ± 3.35	18.44 ± 0.70
MDCN-0.03	1.04 ± 0.05	21.92 ± 0.76	73.14 ± 2.88	16.74 ± 0.24
MDCN-0.05	1.03 ± 0.06	22.33 ± 1.79	69.51 ± 3.84	15.93 ± 0.82
MDCN-0.1	1.02 ± 0.04	21.76 ± 0.74	69.71 ± 3.04	15.54 ± 0.44
